# Comparison of the Structural Characteristics and Functional Performances of Chitosan-Based Active Packaging Films Loaded with Three Varieties of Water Chestnut (*Trapa bispinosa* Roxb.) Shell Powder

**DOI:** 10.3390/foods15183339

**Published:** 2026-09-20

**Authors:** Tianmeng Liao, Bilal Ahmad, Yunlei Wu, Huimin Yong, Juan Kan, Jun Liu

**Affiliations:** College of Food Science and Engineering, Yangzhou University, Yangzhou 225127, China; mz120242276@stu.yzu.edu.cn (T.L.); mh24164@stu.yzu.edu.cn (B.A.); mz120232165@stu.yzu.edu.cn (Y.W.); 008694@yzu.edu.cn (H.Y.); kanjuan@yzu.edu.cn (J.K.)

**Keywords:** antioxidant and antimicrobial activities, fruit peel powder, packaging films, pork preservation, water chestnut

## Abstract

Water chestnut (*Trapa bispinosa* Roxb.) shell is an underutilized fruit processing by-product. Here, three varieties of water chestnut shells (green, red, and black) were pulverized into powder and individually loaded into a chitosan (CS)-based matrix to develop active packaging films via solvent casting. The structural characteristics, physical and functional properties, and minced pork preservation effects of water chestnut shell powder (WCSP)-loaded films were systematically compared with those of the CS film. Results showed that WCSP was rich in lignocellulose and polyphenols. Green and red WCSP with sheet-like morphology were uniformly dispersed in the CS matrix, whereas black WCSP with a bulky, block-like morphology was locally distributed within the matrix. Compared with the CS film, WCSP-loaded films showed higher tensile strength, water vapor permeability, and antioxidant and antimicrobial activities but lower light transmittance, elongation at break, moisture content, water solubility, and swelling ratio. When minced pork was wrapped in CS film and WCSP-loaded films, its shelf life was extended to 4 and 6 days, respectively. Notably, the film incorporated with green WCSP showed the highest antioxidant and antimicrobial activities as well as the optimal pork preservation effect. This study confirms that the water chestnut cultivar significantly affects film properties, providing a new route for high-value utilization of WCSP.

## 1. Introduction

Over the past decades, fossil-derived packaging materials have been extensively applied in the food industry, owing to their low manufacturing cost, outstanding mechanical and barrier properties, as well as favorable thermal processability, printability, and post-processing adaptability. Nevertheless, these non-biodegradable and non-renewable fossil-derived packaging materials pose inherent risks of food-contact substance migration, leading to persistent environmental pollution and chronic food safety hazards [1]. In this context, the development of food packaging materials from biodegradable, renewable, and food-safe ingredients has garnered rapidly growing research attention [2]. Chitosan (CS) is a linear polysaccharide obtained via partial deacetylation of chitin, which is predominantly sourced from crustacean shells and fungal cell walls. The abundant free amino and hydroxyl groups distributed along its backbone endow CS with a unique polyelectrolyte feature and exceptional film-forming capacity [3]. To date, CS has been widely recognized as one of the most promising alternatives to conventional fossil-based substrates for producing food packaging films. Furthermore, a variety of bioactive components are incorporated into CS-based film matrices to achieve tailored functionalities, such as enhanced antioxidant and antimicrobial performances, so as to effectively extend the shelf life of perishable food commodities [4,5].

Food processing by-products are normally generated during the juicing, pulping, and refining of fruits and vegetables. These by-products are conventionally disposed of via incineration and landfilling, which inevitably imposes considerable environmental burdens [6]. Notably, food processing by-products represent an underutilized source rich in high-value polymers (e.g., fiber, starch and proteins) and bioactive substances (e.g., phenolic acids, flavonoids and essential oils), which can be exploited for the fabrication of functional food packaging films [7,8]. In most conventional protocols, target polymers and bioactive substances are first extracted, and then separately applied as film-forming matrices and functional fillers for packaging film production [9]. As an alternative strategy, the pulverized by-product powder can be directly incorporated into polymer matrices to fabricate composite packaging films [10]. This in situ incorporation approach, which introduces the by-product powder into a film-forming system without complex pre-extraction procedures, significantly simplifies the manufacturing process and reduces the overall production cost. To date, a series of food processing by-products including lychee pericarp powder [11], lemon peel powder [12], orange peel powder [13,14], pomegranate peel powder [15], and mangosteen rind powder [16] has been successfully utilized to develop chitosan (CS)-based functional packaging films.

Water chestnut, an aquatic crop belonging to the family Trapaceae, is widely cultivated across European and Asian countries, with a global annual yield exceeding 2 million tons [17]. China stands as the world’s leading water chestnut producer, with its cultivation primarily concentrated in the Yangtze and Pearl River Basins, covering provinces including Jiangsu, Zhejiang, Hubei, Anhui, and Guangdong [18]. The edible fleshy kernel of water chestnut is well recognized for its abundant nutritional components, including starch, dietary fiber, proteins, vitamins, and essential trace elements [19]. Notably, the water chestnut shell, which accounts for approximately 20% of the total fresh fruit weight, is a rich source of lignocellulose and bioactive polyphenols [18,20]. In particular, the abundant polyphenols in water chestnut shells possess antioxidant and antimicrobial activities, making them promising candidates for the fabrication of active packaging films [17]. However, existing research on water chestnut valorization has predominantly focused on the edible fleshy fraction, with particular attention paid to utilizing starch for the fabrication of food packaging films [21,22,23,24,25]. In contrast, the water chestnut shell has rarely been explored as a functional raw material for active packaging film development [26]. Therefore, the present study was designed to valorize the underutilized water chestnut shell for the production of active packaging films, where three varieties of water chestnut shells (green, red, and black) were pulverized into powder and incorporated into a CS-based film-forming matrix. To the best of our knowledge, this work represents the first systematic comparison of the structural characteristics, functional performances, and pork preservation effects of the resultant active packaging films. This study not only achieves the high-value valorization of water chestnut processing waste, but also provides a low-cost, eco-friendly, and sustainable alternative to conventional fossil-derived food packaging films.

## 2. Materials and Methods

### 2.1. Materials and Reagents

Three varieties of fresh water chestnut (*Trapa bispinosa* Roxb.) with distinct shell colors (green, red, and black) were separately purchased from Jiangsu, Anhui, and Hubei provinces (China). All water chestnut samples were manually harvested in September and October 2025. Chitosan (degree of deacetylation: 90%; viscosity: 400 mPa·s), glycerol, 6-hydroxy-2,5,7,8-tetramethylchroman-2-carboxylic acid (Trolox), 2,2-diphenyl-1-picrylhydrazyl (DPPH), and 2,2′-azino-bis(3-ethylbenzothiazoline-6-sulfonic acid) diammonium salt (ABTS) were supplied by Macklin Co. (Shanghai, China). Ultrapure water used in this study was produced by a HyperPureX EUS-13 system (Hyperpurex Instrument Technology Co., Ltd., Shanghai, China). All other reagents were of analytical grade.

### 2.2. Preparation and Proximate Composition Analysis of WCSP

Fresh water chestnut samples were thoroughly rinsed with running tap water before manual shell removal. The shells were carefully separated from the edible flesh of water chestnut and immediately lyophilized. The dried water chestnut shells were pulverized into fine particles and subsequently sieved through a 100-mesh sieve to obtain uniform powder. Three types of WCSP were prepared, namely green water chestnut shell powder (GWCSP), red water chestnut shell powder (RWCSP), and black water chestnut shell powder (BWCSP). All WCSP samples were stored at −80 °C prior to proximate composition analysis and packaging film preparation.

The proximate composition of WCSP samples, including ash, protein, lipid, and crude fiber, was analyzed in accordance with the Chinese National Standard methods GB 5009.4-2016 [27], GB 5009.5-2016 [28], GB 5009.6-2016 [29], and GB/T 5009.10-2003 [30], respectively. Additional functional components in WCSP, including pectin, total polyphenols, total anthocyanins, and total chlorophylls, were quantified using the *m*-hydroxybiphenyl assay [31], Folin-Ciocalteu assay [19], pH-differential assay [32], and spectrophotometric procedure [33], respectively.

### 2.3. Preparation of Active Packaging Films

Active packaging films were prepared via solvent casting [16]. First, CS solution (1.6%, *w*/*v*) was prepared by dissolving CS in the aqueous acetic acid solution (1%, *v*/*v*) under continuous magnetic stirring. Subsequently, three types of WCSP (20%, *w*/*w*, on a dry CS weight basis) were separately added to the CS solution, followed by magnetic stirring for 2 h. Afterwards, glycerol (20%, *w*/*w*, on a dry CS weight basis) was introduced into all film-forming solutions to serve as a plasticizer. Finally, each well-mixed film-forming solution (200 mL) was degassed, cast evenly onto a 24 cm × 24 cm polymethyl methacrylate plate, and dried at 35 °C for 24 h. A total of four films were obtained, namely CS, CS/GWCSP, CS/RWCSP, and CS/BWCSP films. All films were conditioned at 20 °C and 50% relative humidity for 48 h to reach moisture equilibrium before subsequent characterization.

### 2.4. Structural Characterization of WCSP and the WCSP-Loaded Films

The morphology of WCSP and the cross-section of the WCSP-loaded films were observed using a Carl Zeiss GeminiSEM 300 scanning electron microscope (Oberkochen, Germany). The main components of WCSP and the molecular interactions within the films were identified via a Varian 670 Fourier-transform infrared (FT-IR) spectrometer (Palo Alto, CA, USA) over the wavenumber range of 4000–400 cm^−1^. The X-ray diffraction (XRD) patterns of WCSP and the films were recorded on a Bruker D8 Advance X-ray diffractometer (Karlsruhe, Germany) with 2*θ* ranging from 5° to 65°.

### 2.5. Determination of the Physical Properties of the WCSP-Loaded Films

The thickness of the films was evaluated by a Bonthe EVERTE micrometer (Shangqiu, China). The color parameters of the films (L*, a*, b*, and Δ*E*) were measured by a Kangguang SC-80C colorimeter (Beijing, China) with D65 illuminant and 10° observer [11]. The light transmittance of the films was determined by a PerkinElmer Lambda 35 UV-Vis spectrophotometer (Waltham, MA, USA) from 800 to 200 nm [15]. The tensile strength (TS) and elongation at break (EAB) of the films were tested by a Yishite STX200 tensile-testing machine (Yishite, Xiamen, China) with a crosshead speed of 15 mm/min [13]. The thermal degradation of the films was detected by heating samples from 30 to 750 °C in a Henven HTG-1 thermogravimetric (TG) analyzer (Beijing, China) at a heating rate of 10 °C/min [16]. The moisture content (MC) of the films was determined by drying at 105 °C until constant weight [15]. The water solubility (WS) of the films was determined by immersing the absolutely dried films in ultrapure water for 24 h [15]. The swelling ratio (SR) of the films was measured by immersing the conditioned films in ultrapure water for 24 h [11]. The water vapor permeability (WVP) of the films was analyzed by a classic gravimetric method [16].

### 2.6. Determination of the Antioxidant and Antimicrobial Activities of the Films

The antioxidant activity of the films was determined by DPPH and ABTS radical scavenging assays according to the procedure of Jiang et al. [11], using Trolox as a reference antioxidant standard. Results are expressed as μmol Trolox equivalent (TE)/g film. The antimicrobial activity of the films against *Salmonella typhimurium* ATCC 14028 and *Staphylococcus aureus* ATCC 6538 was determined by the disc diffusion method [11]. Briefly, 0.2 mL of 10^6^ CFU/mL bacterial culture was uniformly spread on a lysogeny broth (LB) agar plate. Afterwards, a round film piece (6 mm in diameter) was placed on the upper layer of the plate, and the bacteria were incubated at 37 °C for 12 h. Results are expressed as the diameter of inhibition zones formed around the films.

### 2.7. Food Packaging Test

The minced pork preservation effects of the films were evaluated according to the method of Yun et al. [34]. Fresh minced pork (6 g) was heat-sealed between two pieces of film (6 cm × 6 cm) and stored in sterile Petri dishes at 4 °C for 12 days. The unpackaged minced pork (control group) was directly placed in sterile Petri dishes and stored at 4 °C without any additional wrapping or sealing, fully exposed to the ambient air environment throughout the entire 12-day storage period. The quality indices of minced pork, including total volatile basic nitrogen (TVB-N), thiobarbituric acid reactive substances (TBARS), total viable count (TVC), and sensory attributes, were measured every two days. Specifically, the sensory evaluation of the minced pork was performed in individual booths with controlled light, temperature (20 °C), and humidity (50% relative humidity) by ten trained panelists (5 males and 5 females, aged 20–40 years) from the College of Food Science and Engineering at Yangzhou University. A 5-point descriptive scale (5 = excellent, 4 = good, 3 = acceptable, 2 = unacceptable, 1 = very poor) was applied to assess the odor, color, and overall acceptance of the minced pork.

### 2.8. Statistical Analysis

One-way analysis of variance and the Duncan test were performed using SPSS 13.0 software. Differences were considered statistically significant if *p* < 0.05.

## 3. Results and Discussion

### 3.1. Proximate Composition of Three Varieties of Water Chestnut Shell Powder

To date, the proximate composition of water chestnut kernel has been well documented, with starch, dietary fiber, protein, vitamins, and essential trace elements as its main components [17,19,35]. However, the proximate composition of water chestnut shell has not been systematically investigated yet. As shown in Table 1, three types of WCSP primarily consisted of ash (3.15–5.49%), protein (1.53–4.52%), lipid (3.07–4.56%), crude fiber (17.41–39.46%), and pectin (0.09–0.35%). Notably, the proximate composition of WCSP, especially the crude fiber content, varied significantly with cultivar. The crude fiber fraction in WCSP is mainly attributed to lignocellulose (i.e., cellulose, hemicellulose, and lignin), a tightly cross-linked network in plant cell walls that greatly enhances the mechanical strength of the shell [20]. Notably, BWCSP contained up to 39.46% crude fiber, a value significantly higher than those of GWCSP (17.41%) and RWCSP (21.03%). This discrepancy is mainly because black water chestnut represents the fully mature stage of the fruit, during which its shell is completely lignified with a dense keratinized layer formed on the surface [36]. As a result, the black water chestnut shell exhibits a harder texture than the green and red counterparts. Table 1 also shows that RWCSP had the highest total polyphenol and total anthocyanin contents, followed sequentially by GWCSP and BWCSP. Asha et al. [37] also reported that RWCSP possessed a higher polyphenol content than GWCSP. Liu et al. [18] demonstrated that the typical polyphenolic compounds in WCSP included pyrogallol, gallic acid, methyl gallate, 2,4,6-trihydroxybenzoic acid, 5-hydroxyisovanillic acid, caffeic acid, naringenin, phloretin, catechin, gallocatechin, silybin and silydianin. In addition, GWCSP exhibited the highest total chlorophyll content, enabling the shell to present a characteristic green color.

### 3.2. Structural Characteristics of Three Varieties of Water Chestnut Shell Powder

**Figure 1:**
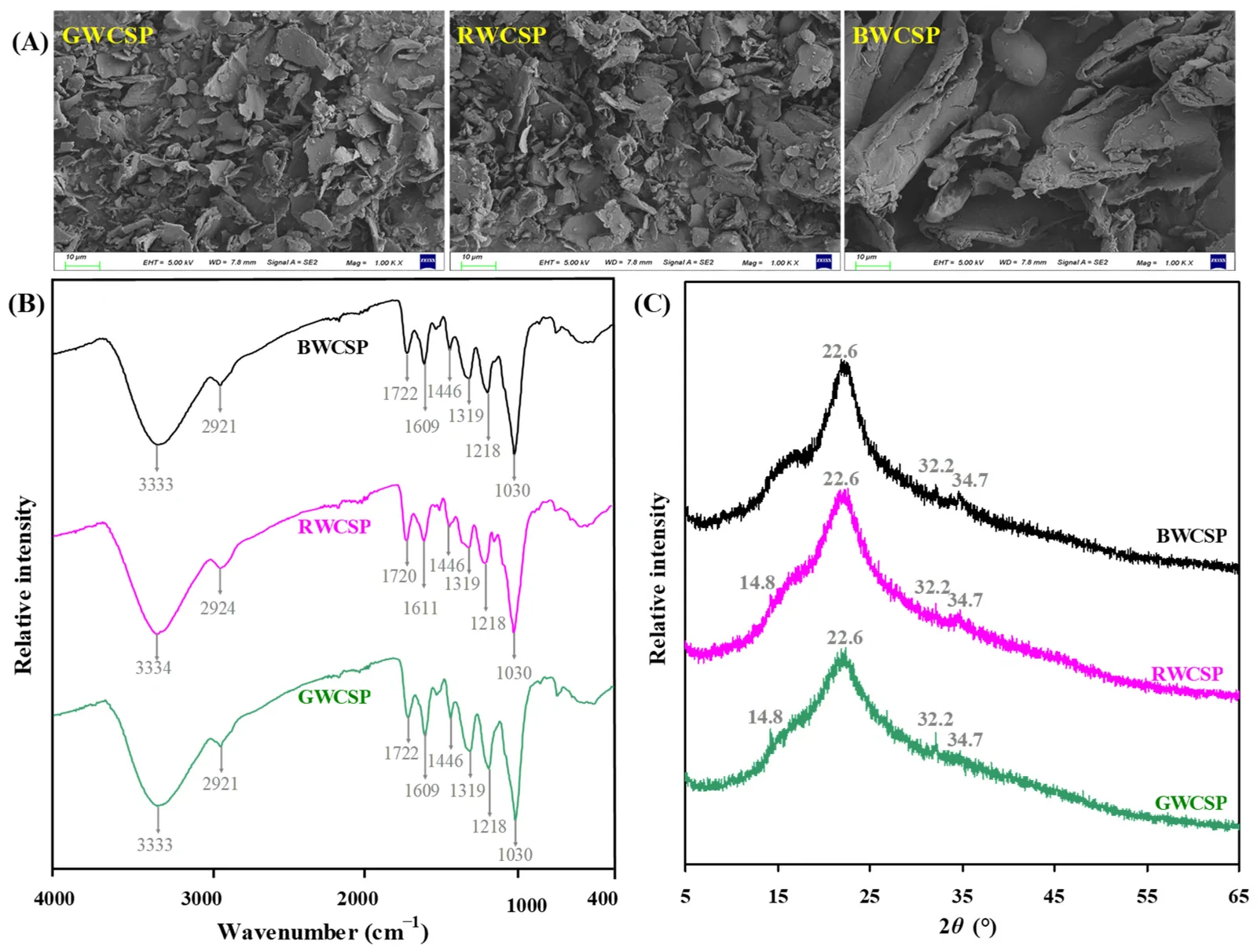
SEM micrographs (**A**), FT-IR spectra (**B**) and XRD patterns (**C**) of GWCSP, RWCSP and BWCSP.

The structural characteristics of the three types of WCSP were characterized using SEM, an FT-IR spectrometer, and an X-ray diffractometer. As illustrated in Figure 1A, both GWCSP and RWCSP exhibited typical sheet-like morphology with fine fragmented debris attached, while BWCSP presented an entirely different bulky block morphology. Such distinct morphological discrepancies among the three WCSP samples are attributed to their significant differences in variety and physiological maturity. Specifically, green water chestnut and red water chestnut are at the immature and semi-mature stages, respectively, where the lignification process of their shells remains incomplete, accompanied by relatively lower crude fiber contents (Table 1). Under this condition, their shell tissues are fragile and prone to being crushed into thin sheet-like fragments during pulverization. In contrast, black water chestnut reaches the full maturity stage, and its shell has undergone thorough lignification with a remarkably higher crude fiber content (Table 1). This physiological trait endows BWCSP with a compact and rigid texture that is highly resistant to mechanical pulverization, thus forming the bulky block morphology [36].

The FT-IR spectra of the three types of WCSP are highly similar (Figure 1B). The bands around 3333 and 2921 cm^−1^ were attributed to O–H stretching vibration of hydroxyl groups and C–H stretching vibration of aliphatic methylene groups, respectively. The characteristic band around 1722 cm^−1^ was assigned to the C=O stretching vibration of carbonyl groups in hemicellulose and lignin [38]. Notably, two other characteristic bands around 1609 and 1446 cm^−1^ were assigned to C=C stretching vibrations of aromatic rings in lignin [39]. The band at 1218 cm^−1^ corresponded to C–O stretching vibration of guaiacyl units in lignin [40]. The bands at 1319 and 1030 cm^−1^ corresponded to –CH_2_ scissoring and C–O–C stretching vibrations, respectively, in cellulose and hemicellulose [41].

The XRD patterns of three types of WCSP are shown in Figure 1C. All WCSP samples displayed a major diffraction peak at 22.6° and a weak diffraction peak at 34.7°, corresponding to the (200) and (004) planes of the cellulose I structure, respectively [42]. Another weak diffraction peak, corresponding to the (1–10) plane of cellulose I, was noted at 14.8° for GWCSP and RWCSP but was absent in BWCSP [43]. The typical (110) plane of the cellulose I structure, which normally appears at 16.5°, was not observed in any WCSP samples. This might be because the ordered semi-crystalline structure of cellulose was partially disrupted by lignin and hemicellulose [43]. Notably, BWCSP exhibited a broad amorphous halo centered around 14–18°, which might originate from amorphous components, such as lignin, hemicellulose, and non-crystalline cellulose regions. This further demonstrated that BWCSP was highly lignified.

### 3.3. Structural Characteristics of the Films

**Figure 2:**
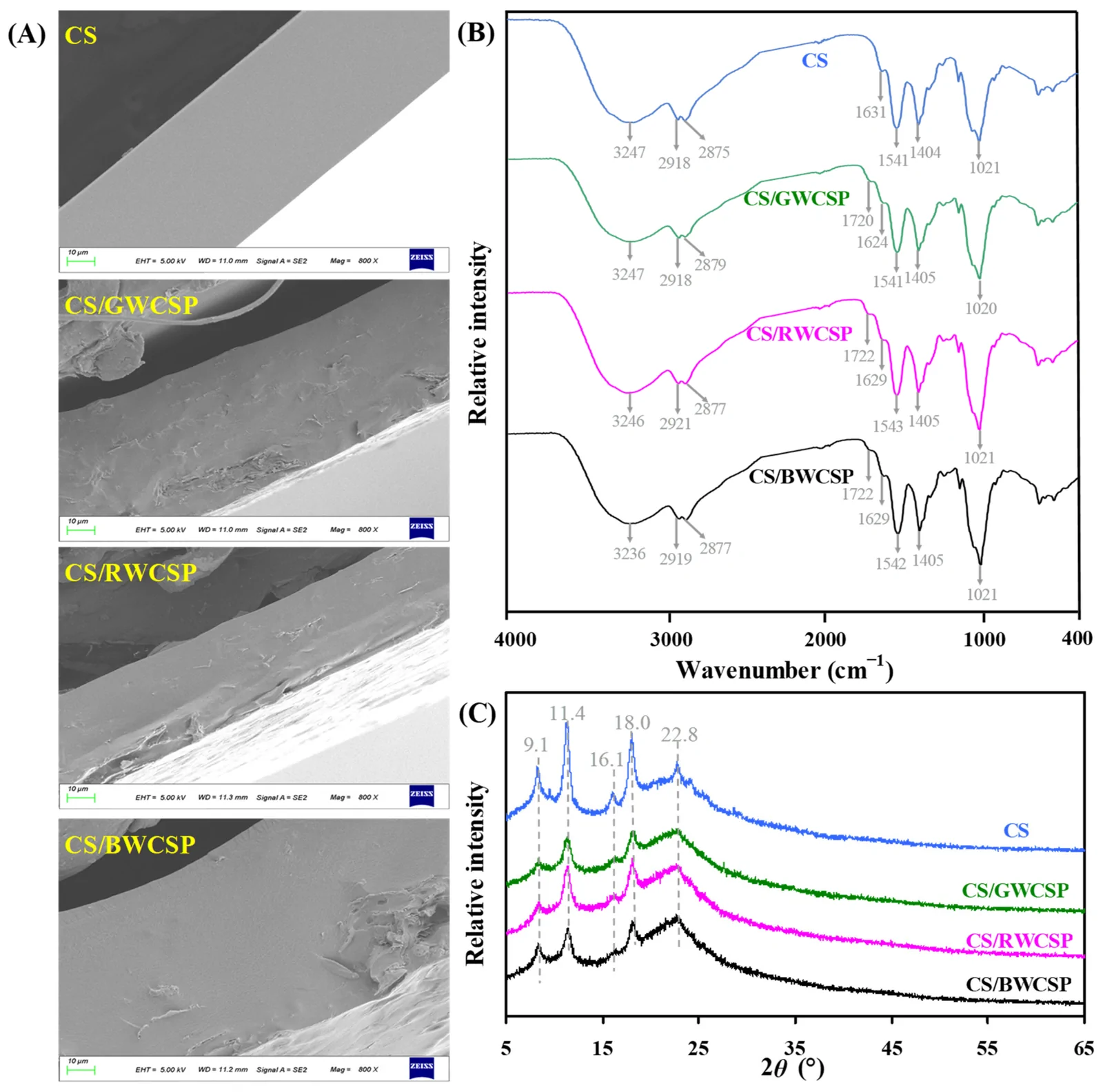
Cross-sectional morphology (**A**), FT-IR spectra (**B**), and XRD patterns (**C**) of CS, CS/GWCSP, CS/RWCSP, and CS/BWCSP films.

Four kinds of packaging films, namely CS, CS/GWCSP, CS/RWCSP, and CS/BWCSP films, were prepared, and their structural characteristics were characterized using SEM, an FT-IR spectrometer, and an X-ray diffractometer. As illustrated in Figure 2A, the CS film exhibited a smooth and dense cross-section, owing to the high compatibility between CS and glycerol [15,16]. In contrast to the CS film, all the WCSP-loaded films exhibited heterogeneous morphologies with rough cross-sections. Notably, GWCSP and RWCSP with sheet-like morphology were uniformly dispersed in the CS matrix, while BWCSP with a bulky block morphology was locally distributed within the matrix. Nonetheless, all the WCSP-loaded films still maintained dense and compact microstructures, suggesting the formation of favorable interfacial adhesion between the CS matrix and WCSP fillers. In general, the microstructures of fruit peel powder-loaded films are influenced by the source of fruit peel, the particle size and loading content of peel powder, and film preparation conditions [10]. Previous studies have demonstrated that the microstructure of CS/fruit peel powder films is mainly governed by the powder loading content [11,15,16]. In the present study, our results further reveal that the microstructure of CS/WCSP films is significantly modulated by the cultivar of WCSP.

As presented in Figure 2B, the FT-IR spectra of the CS film exhibited the characteristic absorption bands of CS at 3247 cm^−1^ (O–H and N–H stretching vibration), 2918 and 2875 cm^−1^ (C–H stretching vibration), 1631 cm^−1^ (C=O stretching vibration), 1541 cm^−1^ (N–H bending vibration), 1404 cm^−1^ (C–H bending vibration), and 1021 cm^−1^ (C–O–C stretching vibration) [11,16]. A new absorption band was noted in the WCSP-loaded films around 1722 cm^−1^, assigned to C=O stretching of carbonyl groups in hemicellulose and lignin [38]. This confirmed the successful loading of different types of WCSP. Notably, the characteristic absorption bands of CS slightly shifted in all the WCSP-loaded films, which was caused by the intermolecular interactions (e.g., hydrogen bonds, electrostatic interactions, and van der Waals forces) between the CS matrix and WCSP fillers [11,15,16]. These non-covalent intermolecular interactions did not destroy the basic backbone structure of CS, but significantly improved the interfacial compatibility between the CS matrix and WCSP fillers.

The CS film exhibited a semi-crystalline character, with five typical diffraction peaks of CS at 9.1°, 11.4°, 16.1°, 18.0°, and 22.8° (Figure 2C) [44]. The semi-crystalline character of the CS film is attributed to strong intramolecular and intermolecular hydrogen bonds of CS chains [11]. Although all the WCSP-loaded films retained the diffraction peaks of CS, their peak intensities were much lower than those of the CS film. This indicated that the CS-WCSP interfacial interactions interfered with the formation of an ordered semi-crystalline conformation in CS. Other researchers also observed reduced crystallinity in CS films after loading lychee pericarp powder [11] and pomegranate peel powder [15].

### 3.4. Physical Properties of the Films

#### 3.4.1. Thickness, Color and Light Transmittance

As shown in Table 2, the thickness of the CS film (0.077 mm) increased to 0.111–0.128 mm after the loading of WCSP. This was because the solid WCSP fillers with certain particle sizes were physically embedded in the CS matrix, which introduced extra solid volume [11,16]. Meanwhile, the strong intermolecular interactions formed between CS and WCSP fillers interfered with the ordered arrangement of CS chains, which loosened the originally compact network structure of the CS film. Notably, the CS/BWCSP film had a greater thickness than the CS/GWCSP and CS/RWCSP films, which was attributed to the fact that BWCSP possessed a larger average particle size than GWCSP and RWCSP (Figure 1A).

**Figure 3:**
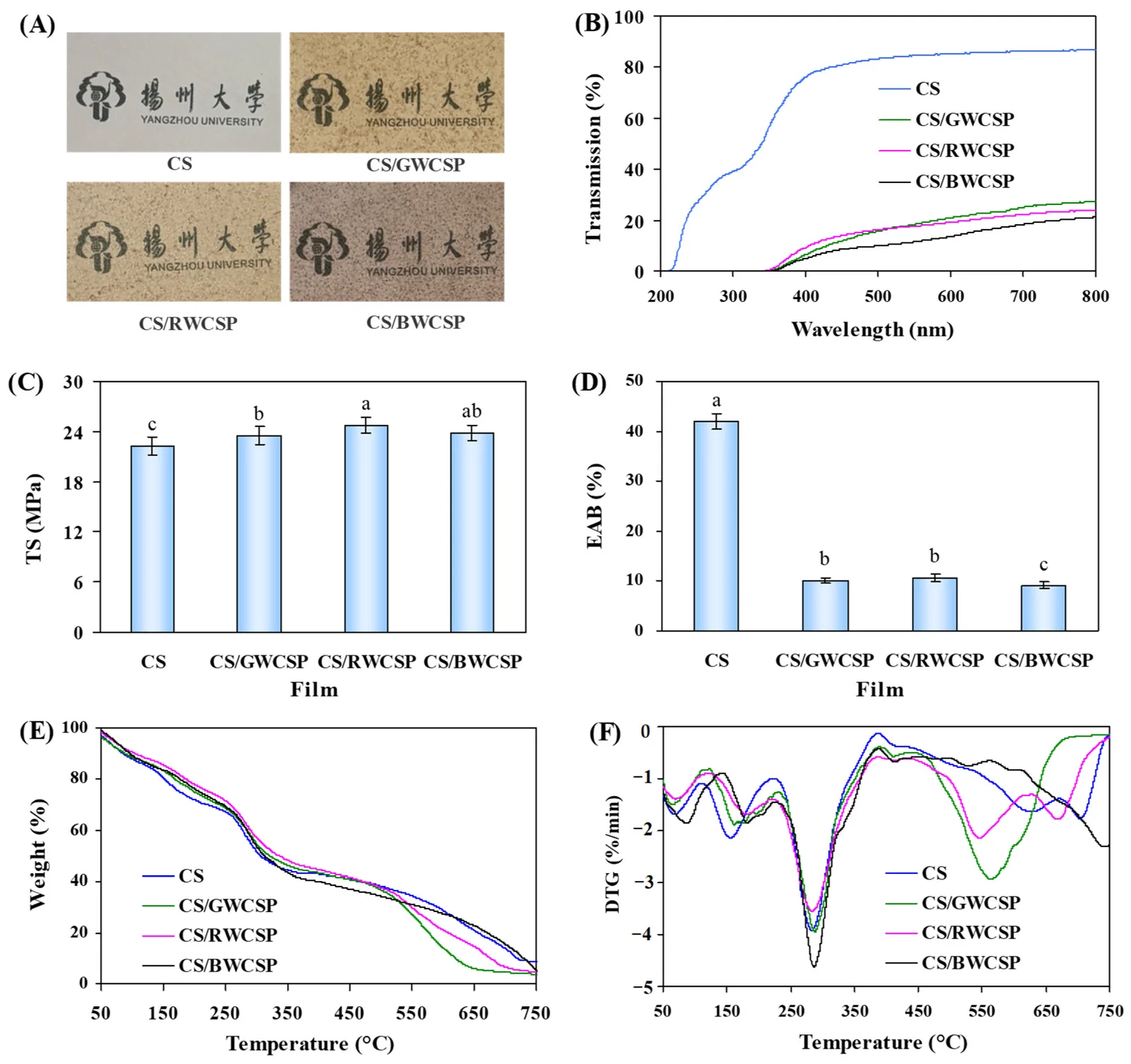
Appearances (**A**), light transmittance (**B**), TS (**C**), EAB (**D**), TG (**E**) and DTG (**F**) profiles of CS, CS/GWCSP, CS/RWCSP, and CS/BWCSP films. Each value represents the mean ± SD (*n* = 3 for light transmittance and *n* = 10 for TS and EAB). Different lowercase letters indicate statistically significant differences (*p* < 0.05) among different films.

As illustrated in Figure 3A, the loading of WCSP made the colorless CS film turn brown or tan. Notably, the color of the WCSP-loaded films was not consistent with that of water chestnut shell. This distinct color discrepancy could be explained by the distribution characteristics of pigments in raw water chestnut shell. The natural pigments (e.g., polyphenols, anthocyanins, and chlorophylls) are normally localized in the outer epidermal layer of water chestnut shell, which only accounts for an extremely low proportion in the whole WCSP (Table 1). During the film-forming process, these limited epidermal pigments were homogeneously dispersed in the CS matrix, thus further diluting the coloration effect of the pigments. Previous studies also reported that CS films loaded with fruit peel powder showed a brown color [11,15,16]. The color parameters of the films (L*, a*, b*, and Δ*E*) in Table 2 further demonstrated the color differences among the WCSP-loaded films.

The light transmittance of the films in Figure 3B shows that the transparency of the CS film was reduced by WCSP. This was because the insoluble WCSP in the CS matrix acted as heterogeneous light scattering centers, which prevented light from passing through the films [11,16]. Meanwhile, the chromophores in WCSP components (e.g., polyphenols, anthocyanins, chlorophylls, and lignin) were capable of absorbing UV-vis light, thereby reducing the light transmittance of the resultant films [11,15,16]. Notably, the CS/BWCSP film showed relatively lower transparency than the CS/GWCSP and CS/RWCSP films. This was probably because BWCSP had a larger average particle size that could more effectively block and scatter light. The light transmittance of the WCSP-loaded films was significantly lower than that of the previously reported CS films containing lychee pericarp powder [11], pomegranate peel powder [15], and mangosteen rind powder [16]. This reveals that the WCSP-loaded films have strong light-barrier ability that can be used to inhibit light-induced food spoilage.

#### 3.4.2. Mechanical Properties

The mechanical properties of the films were measured using a tensile-testing machine, and their TS and EAB are shown in Figure 3C and Figure 3D, respectively. The TS of the CS film reached 22.28 MPa, which was correlated with the molecular weight and the degree of deacetylation of CS as well as the content of glycerol [45]. After the loading of three types of WCSP fillers, the TS of the CS film was enhanced to 23.55–24.71 MPa. Such minor TS improvements were attributed to the intermolecular interactions (e.g., hydrogen bonds, electrostatic interactions, and van der Waals forces) between the CS matrix and WCSP fillers, which generated an interfacial adhesion effect [11,16]. Previous studies have also reported that the incorporation of lychee pericarp powder [11] and mangosteen rind powder [16] effectively enhanced the TS of CS films. Among all the WCSP-loaded films, the CS/RWCSP film showed the highest TS, owing to the highest soluble pectin content in RWCSP (Table 1), which could intertwine with CS chains via hydrogen bonds and electrostatic interactions [46]. The EAB of the CS film decreased from 41.95% to 9.17–10.64% after the loading of WCSP, indicating that the flexibility of the CS film was significantly reduced by WCSP. This phenomenon was mainly caused by the disruption of film continuity induced by the insoluble components in WCSP, which was consistent with previous reports that lychee pericarp powder [11] and mangosteen rind powder [16] could also significantly reduce the EAB of CS films. The sharp drop in EAB and consequent embrittlement of WCSP-loaded CS films raises clear practical barriers for industrial packaging applications. This brittleness increases the risk of fracture during high-speed slitting, heat sealing, and machine handling. To address this limitation, some feasible strategies are proposed for future studies. For instance, the plasticizer content in the films can be optimized to balance TS and EAB. Alternatively, reducing WCSP particle size via superfine grinding is helpful to minimize matrix discontinuity.

#### 3.4.3. Thermal Property

The thermal stability of the films was characterized using a TG analyzer, and their TG and derivative thermogravimetric (DTG) profiles are displayed in Figure 3E and Figure 3F, respectively. The thermal degradation of the CS film consisted of four stages, including moisture evaporation (50–104 °C), glycerol degradation (105–225 °C), CS backbone pyrolysis (226–404 °C), and residual carbonization (405–750 °C) [16]. Similar to the CS film, all WCSP-loaded films also exhibited a four-stage thermal degradation behavior. Notably, the CS film showed a faster degradation rate than WCSP-loaded films at the first and second stages, implying a relatively higher initial MC in the CS film. Among the three types of WCSP fillers, BWCSP with the largest average particle size presented the most prominent barrier effect, thus endowing the CS/BWCSP film with the slowest mass loss rates of moisture and glycerol. However, all WCSP-loaded films degraded more rapidly than the CS film at the fourth stage. This was mainly because the loaded WCSP introduced abundant micro-pores and structural defects in the formed char layer, which could not effectively block the outward diffusion of pyrolysis products. Zhang et al. [16] found that the thermal degradation behavior of the CS film was not significantly affected by the incorporation of 2.5%, 5%, and 10% of mangosteen rind powder. This suggests that obvious thermal property changes can only be observed when the added powder reaches a specific concentration in the film matrix.

#### 3.4.4. MC, WS, SR, and WVP

**Figure 4:**
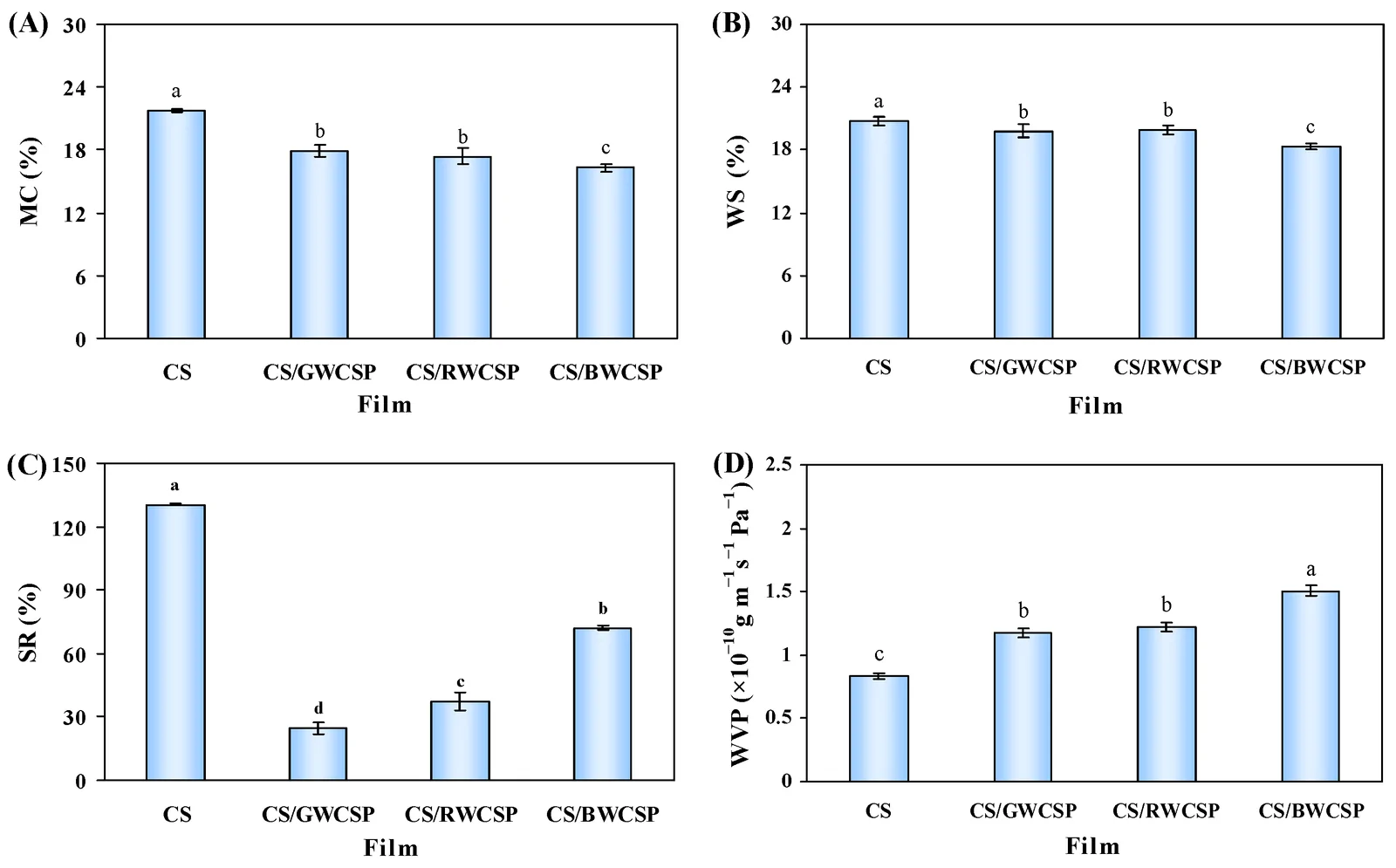
MC (**A**), WS (**B**), SR (**C**), and WVP (**D**) of CS, CS/GWCSP, CS/RWCSP, and CS/BWCSP films. Each value represents the mean ± SD (*n* = 3). Different lowercase letters indicate statistically significant differences (*p* < 0.05) among different films.

As illustrated in Figure 4A,B, both the MC and WS of the CS film slightly decreased after the loading of three types of WCSP fillers. The high MC and WS of the CS film originated from the inherent hygroscopic characteristics of the CS matrix and glycerol [15]. In contrast, the relatively lower MC and WS of the WCSP-loaded films were attributed to the abundant hydrophobic lignocellulose fractions in WCSP [16]. Among these WCSP-loaded films, the CS/BWCSP film exhibited the lowest MC and WS, which was consistent with its highest lignocellulose proportion (Table 1). In addition, the interfacial adhesion force formed between the CS matrix and WCSP fillers further reduced the exposure of hydrophilic groups on CS chains, thereby effectively lowering the MC and WS of the resultant films [11,16]. Other researchers also observed that the MC and WS of CS films decreased after lychee pericarp powder [11] and mangosteen rind powder [16] were incorporated into the films.

The SR of the CS film decreased from 130.58% to 24.54–72.09% after the loading of three types of WCSP fillers (Figure 4C). The high SR of the CS film originated from the abundant hydrophilic hydroxyl and amino groups on its molecular chains, which could form massive hydrogen bonds with water molecules [47]. Meanwhile, water molecules could penetrate into the film matrix and break hydrogen bonds, further promoting the water absorption and volume expansion of the film. The glycerol plasticizer possesses strong hygroscopicity and can also enhance the SR of the CS film. The significantly lower SR of the WCSP-loaded films was attributed to hydrophobic lignocellulose in WCSP, which weakened the water affinity of the CS matrix [11]. Meanwhile, the interfacial adhesion force formed between WCSP fillers and the CS matrix reduced the number of free hydrophilic sites for water binding, and the resultant compact WCSP-filled network further restricted the volume expansion of the films in water [11]. Among these WCSP-loaded films, the CS/BWCSP film exhibited the highest SR. This was because BWCSP, with a bulky block structure, was locally distributed within the matrix (Figure 2A), which had a weaker impact on the SR of the CS film.

The WVP of the films was determined via a standard gravimetric method. As presented in Figure 4D, the WVP of the CS film was 0.83 × 10^−10^ g m^−1^ s^−1^ Pa^−1^, which was remarkably lower than those of the WCSP-loaded films (1.17–1.51 × 10^−10^ g m^−1^ s^−1^ Pa^−1^). This was because WCSP broke the continuous compact structure of the CS matrix (Figure 2A), and the interfacial gaps between WCSP fillers and the CS matrix provided extra channels for water vapor transmission [16]. Other researchers have demonstrated that the WVP of CS-based films progressively increases with the elevation of fruit peel powder contents [15,16]. Among all the WCSP-loaded films, the CS/BWCSP film exhibited the highest WVP, which was because the large-sized BWCSP created a wide interfacial gap for efficient water vapor diffusion. Notably, this moderate increase in WVP is favorable for food preservation applications, as it prevents excessive moisture accumulation inside the package and effectively extends the shelf life of high-moisture packaged foods [48]. Despite this, a low WVP is normally required to restrict moisture transfer between the packaged food and the surrounding environment. In future studies, the WVP of WCSP-loaded films can be reduced by reducing WCSP particle size, introducing a thin hydrophobic surface layer, and incorporating additional cross-linking agents.

### 3.5. Functional Properties of the Films

#### 3.5.1. Antioxidant Activity

**Figure 5:**
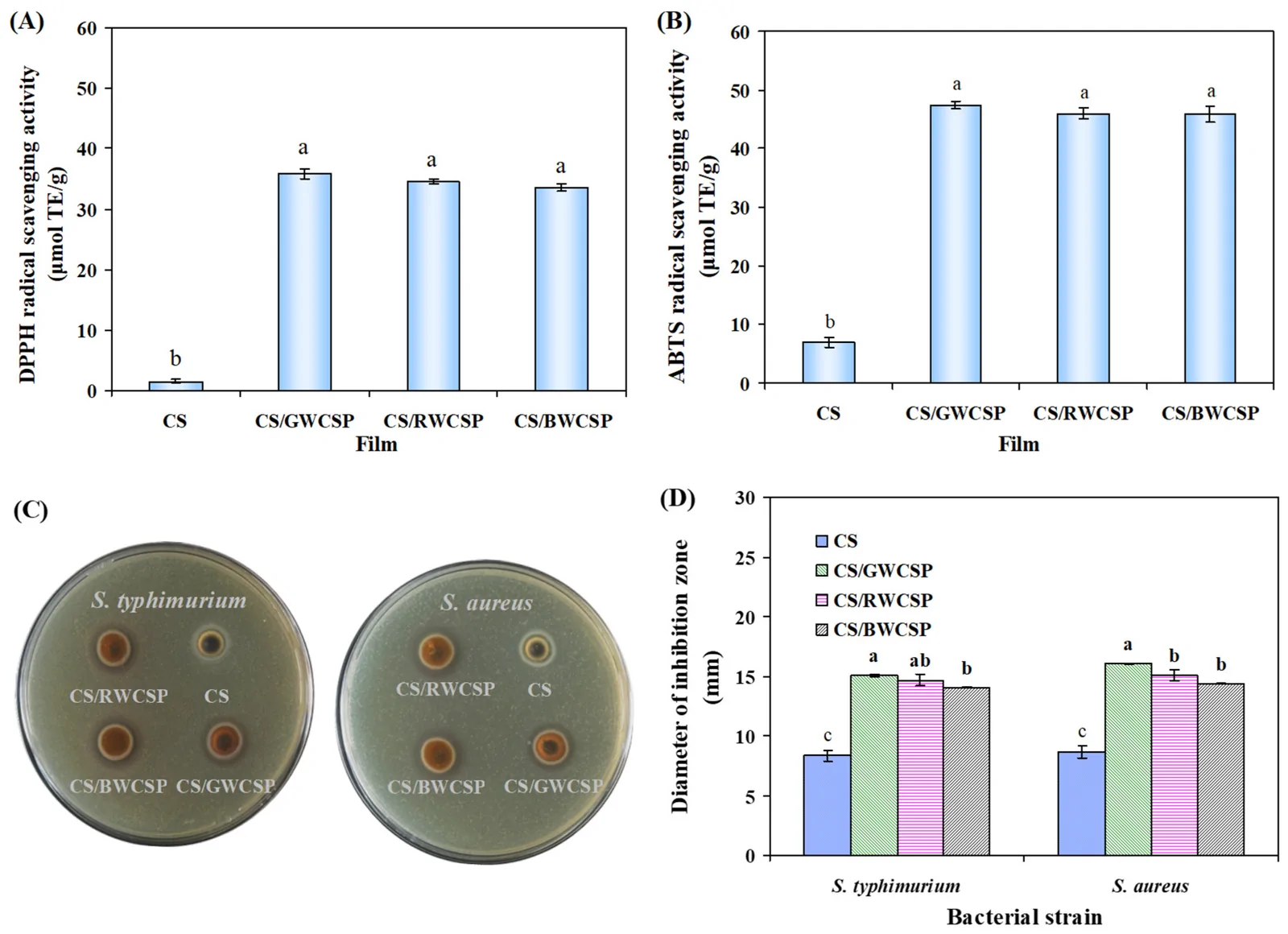
DPPH (**A**) and ABTS (**B**) radical scavenging activities of CS, CS/GWCSP, CS/RWCSP, and CS/BWCSP films. Antimicrobial performance (**C**) and inhibition zone diameters formed (**D**) of CS, CS/GWCSP, CS/RWCSP, and CS/BWCSP films against *S. typhimurium* and *S. aureus*. Each value represents the mean ± SD (*n* = 3). Different lowercase letters indicate statistically significant differences (*p* < 0.05) among different films.

The antioxidant activity of the films was evaluated via DPPH and ABTS scavenging assays, and the corresponding results are presented in Figure 5A and Figure 5B, respectively. All results are expressed as TE, where a higher TE value indicates a stronger free radical scavenging activity. The CS film exhibited low DPPH and ABTS scavenging activities, which were associated with the limited free amino groups in the CS matrix [13]. Compared with the CS film, the WCSP-loaded films showed higher DPPH and ABTS scavenging activities, which were attributed to the abundant inherent antioxidants in WCSP, including polyphenols, anthocyanins, and chlorophylls (Table 1). The superior antioxidant activity of the WCSP-loaded films enables them to effectively inhibit food oxidation in practical food packaging applications. Many researchers also found that the antioxidant activity of CS-based films was elevated by incorporating fruit peel powders, such as lychee pericarp powder [11], lemon peel powder [12], orange peel powder [13], pomegranate peel powder [15], and mangosteen rind powder [16]. The DPPH radical scavenging activities of CS/GWCSP, CS/RWCSP, and CS/BWCSP films were 35.76, 34.67, and 33.52 μmol TE/g, respectively, while their ABTS radical scavenging activities were 47.43, 46.01, and 45.86 μmol TE/g, respectively. The minor differences in the antioxidant activity of the WCSP-loaded films were mainly attributed to the fact that the three types of WCSP had different contents of polyphenols, anthocyanins, and chlorophylls (Table 1).

#### 3.5.2. Antimicrobial Activity

The antimicrobial performances of the films against Gram-negative *S. typhimurium* and Gram-positive *S. aureus* are presented in Figure 5C. The corresponding quantitative results, expressed as the diameters of the inhibition zones formed around each film sample, are summarized in Figure 5D. The CS film possessed intrinsic antimicrobial activity, which could be attributed to the protonated amino groups along the CS backbone that interacted with bacterial cell membranes, leading to the disruption of the membrane barrier and the leakage of intracellular components [15,16]. As expected, all WCSP-loaded films showed significantly higher antimicrobial activities than the CS film. This enhancement was mainly because the abundant polyphenols in WCSP were potent antimicrobials that could destroy the bacterial outer membrane barrier, penetrate into the intracellular matrix to inactivate key metabolic enzymes, and eventually achieve a remarkable synergistic bacteriostatic effect with CS [15,16]. In addition, the chlorophylls in WCSP also possessed certain antimicrobial activity, while their underlying action mechanisms are still not fully elucidated [49]. Given that GWCSP had the highest total chlorophyll content and a comparable total phenol content among the three WCSP samples, the CS/GWCSP film presented the strongest antimicrobial activity. Previous studies have demonstrated that the incorporation of different fruit peel powders, including lychee pericarp powder [11], lemon peel powder [12], pomegranate peel powder [15], and mangosteen rind powder [16], increases the antimicrobial activity of CS-based films. In the present study, the excellent antimicrobial performance of the WCSP-loaded films endows them with great application potential to inhibit microbial proliferation in practical food packaging scenarios.

### 3.6. Application of the Films in Pork Preservation

**Figure 6:**
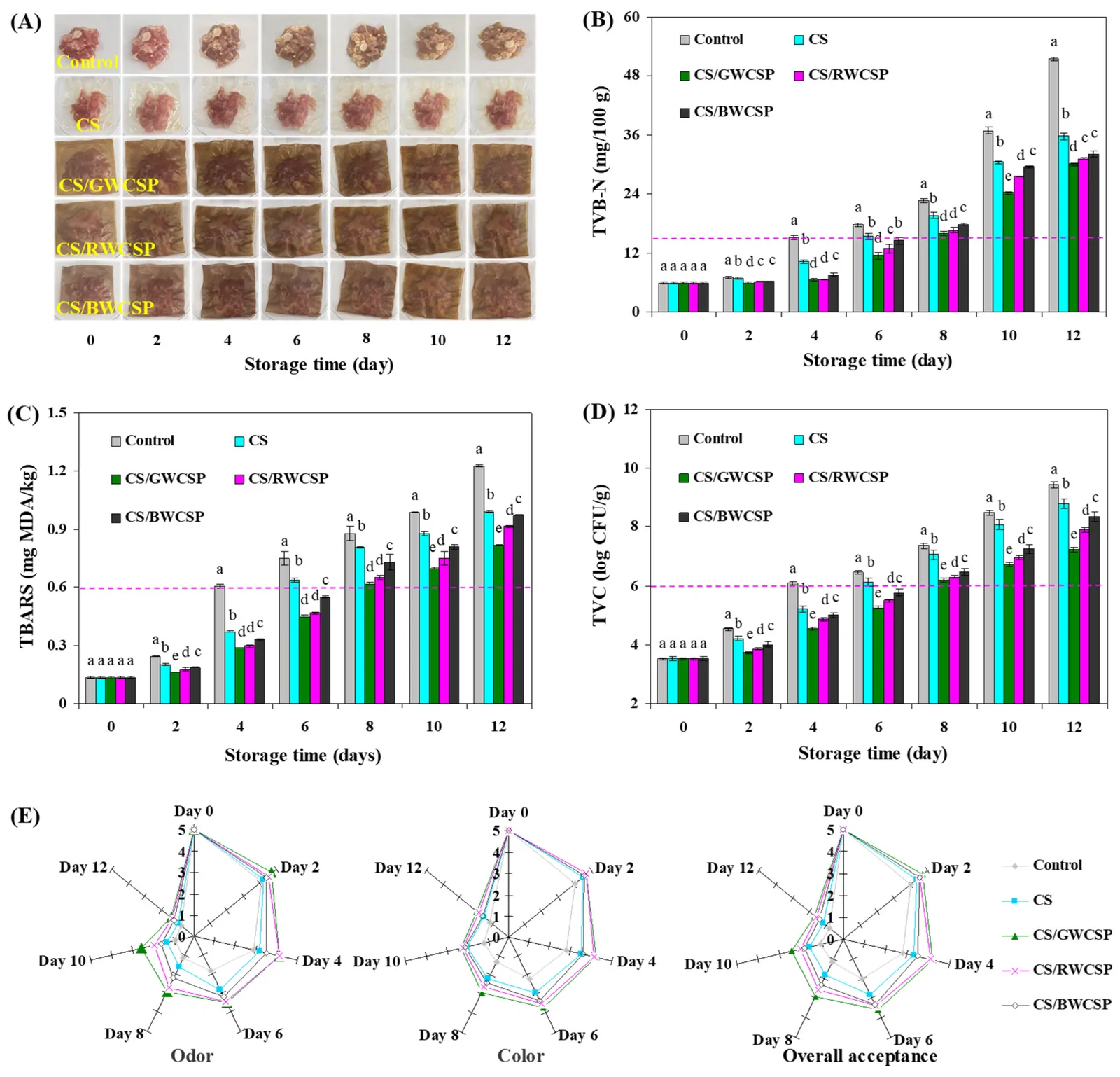
Appearances (**A**), TVB-N (**B**), TBARS (**C**), TVC (**D**), and sensory attributes (**E**) of minced pork in different treatment groups during storage. Each value represents the mean ± SD (*n* = 3 for TVB-N, TBARS, and TVC; *n* = 10 for odor, color, and overall acceptance). The dashed lines in Panels (**B**–**D**) represent the maximum permissible limits of TVB-N, TBARS and TVC for minced pork, respectively. Different lowercase letters indicate statistically significant differences (*p* < 0.05) among different treatment groups.

Given that the developed films had potent antioxidant and antimicrobial activities, they were further applied to package minced pork. As illustrated in Figure 6A, the color of minced pork in the control group (without film packaging) turned dark from the fourth day, indicating that the quality of the meat sample deteriorated very rapidly. However, minced pork packaged with CS film retained its original color for 12 days. The quantitative quality indicators of minced pork, including TVB-N, TBARS, and TVC, are presented in Figure 6B, Figure 6C and Figure 6D, respectively. These three parameters are widely accepted as standard indicators used to evaluate protein decomposition, lipid oxidation, and microbial contamination in meat systems, respectively [30]. The results showed that the levels of TVB-N, TBARS, and TVC gradually increased with the extension of minced pork preservation time. According to Chinese National Standard GB/T 9959.2-2008 [50], the maximum permissible limits for fresh pork are 15 mg/100 g for TVB-N, 0.6 mg MDA/kg for TBARS, and 6 log CFU/g for TVC, respectively. Based on these criteria, minced pork in the control group became non-fresh on the fourth day, which was consistent with its remarkable color changes. As compared with pork in the control group, pork wrapped in CS film exhibited a significantly slower quality deterioration rate, extending the shelf life of minced pork from 2 to 4 days. Notably, WCSP-loaded films exhibited better preservation effects on minced pork than the CS film. Among all WCSP-loaded films, the CS/GWCSP film showed the optimal pork preservation effect, followed by the CS/RWCSP film and the CS/BWCSP film. This trend was consistent with the antioxidant and antimicrobial activities of WCSP-loaded films (Figure 5). The shelf life of minced pork packaged with all WCSP-loaded films was extended to 6 days. The sensory attributes (color, odor, and overall acceptance) of minced pork in different treatment groups are shown in Figure 6E, which further verified the excellent preservation efficacy of WCSP-loaded films for minced pork. Previous studies have demonstrated that CS-based films incorporated with fruit peel powder are effective in extending the shelf life of fresh-cut apple [11], blueberries [12], and soybean oil [16]. The present study further demonstrates the feasibility of applying WCSP-loaded films in pork preservation, which can be attributed to the excellent barrier, antioxidant, and antimicrobial properties of the films.

## 4. Conclusions

This study successfully utilized water chestnut shell for the fabrication of CS-based active packaging films. Notably, the proximate composition of WCSP, especially the crude fiber content, varied significantly with cultivar. Structural characterization revealed that the crude fiber in WCSP was mainly composed of lignocellulose. The size of WCSP greatly affected the cross-sectional morphology, light transmittance, thermal stability, and hydrophobicity of the films. The total polyphenol and chlorophyll contents in WCSP significantly influenced the antioxidant and antimicrobial activities of the films. Among the three WCSP-loaded films, the CS/GWCSP film presented the highest antioxidant and antimicrobial activities. The shelf life of minced pork was extended to 6 days by the WCSP-loaded films. In future studies, the physical properties of the WCSP-loaded films, especially EAB and WVP, should be optimized to enhance industrial viability.

## Figures and Tables

**Table 1:** Proximate composition of GWCSP, RWCSP and BWCSP.

Proximate Composition	GWCSP	RWCSP	BWCSP
Ash (%)	5.49 ± 0.06 ^a^	3.85 ± 0.05 ^b^	3.15 ± 0.05 ^c^
Protein (%)	4.52 ± 0.21 ^a^	3.13 ± 0.27 ^b^	1.53 ± 0.22 ^c^
Lipid (%)	3.07 ± 0.13 ^b^	3.11 ± 0.25 ^b^	4.56 ± 0.09 ^a^
Crude fiber (%)	17.41 ± 1.29 ^c^	21.03 ± 1.07 ^b^	39.46 ± 3.55 ^a^
Pectin (%)	0.19 ± 0.01 ^b^	0.35 ± 0.05 ^a^	0.09 ± 0.05 ^c^
Total polyphenols (mg GAE/100 g powder)	16.86 ± 0.04 ^a^	17.84 ± 0.04 ^a^	10.66 ± 0.03 ^b^
Total anthocyanins (mg CGE/100 g powder)	0.80 ± 0.03 ^b^	2.86 ± 0.07 ^a^	0.13 ± 0.03 ^c^
Total chlorophylls (mg/100 g powder)	127.64 ± 2.93 ^a^	25.45 ± 1.99 ^b^	26.63 ± 0.66 ^b^

Each value represents the mean ± SD (*n* = 3). Different lowercase letters in the same row indicate significant differences (*p* < 0.05).

**Table 2:** Thickness and color parameters (L*, a*, b*, and Δ*E*) of CS films loaded with GWCSP, RWCSP, and BWCSP.

Films	Thickness (mm)	L*	a*	b*	Δ*E*
CS	0.077 ± 0.014 ^c^	87.85 ± 0.53 ^a^	1.23 ± 0.03 ^d^	−2.54 ± 0.01 ^d^	5.20 ± 0.02 ^c^
CS/GWCSP	0.111 ± 0.005 ^b^	68.59 ± 0.36 ^b^	5.24 ± 0.26 ^c^	19.46 ± 0.15 ^a^	34.66 ± 0.36 ^a^
CS/RWCSP	0.114 ± 0.005 ^b^	65.56 ± 0.13 ^c^	6.01 ± 0.08 ^b^	14.37 ± 0.14 ^b^	33.66 ± 0.15 ^b^
CS/BWCSP	0.128 ± 0.004 ^a^	62.89 ± 0.05 ^d^	9.10 ± 0.21 ^a^	8.82 ± 0.04 ^c^	33.60 ± 0.10 ^b^

Values are given as mean ± SD (*n* = 10 for thickness, *n* = 3 for color parameters). Different lowercase letters in the same column indicate significant differences (*p* < 0.05).

## Data Availability

The original contributions presented in this study are included in the article. Further inquiries can be directed to the corresponding author.
